# Molecular networking in the neuronal ceroid lipofuscinoses: insights from mammalian models and the social amoeba *Dictyostelium discoideum*

**DOI:** 10.1186/s12929-020-00653-y

**Published:** 2020-05-20

**Authors:** Robert J. Huber

**Affiliations:** Department of Biology, Trent University, 1600 West Bank Drive, Peterborough, Ontario K9L 0G2 Canada

**Keywords:** Neuronal ceroid lipofuscinosis, Batten disease, *Dictyostelium discoideum*, Molecular networking, Neurodegeneration

## Abstract

The neuronal ceroid lipofuscinoses (NCLs), commonly known as Batten disease, belong to a family of neurological disorders that cause blindness, seizures, loss of motor function and cognitive ability, and premature death. There are 13 different subtypes of NCL that are associated with mutations in 13 genetically distinct genes (*CLN1-CLN8*, *CLN10-CLN14*). Similar clinical and pathological profiles of the different NCL subtypes suggest that common disease mechanisms may be involved. As a result, there have been many efforts to determine how NCL proteins are connected at the cellular level. A main driving force for NCL research has been the utilization of mammalian and non-mammalian cellular models to study the mechanisms underlying the disease. One non-mammalian model that has provided significant insight into NCL protein function is the social amoeba *Dictyostelium discoideum*. Accumulated data from *Dictyostelium* and mammalian cells show that NCL proteins display similar localizations, have common binding partners, and regulate the expression and activities of one another. In addition, genetic models of NCL display similar phenotypes. This review integrates findings from *Dictyostelium* and mammalian models of NCL to highlight our understanding of the molecular networking of NCL proteins. The goal here is to help set the stage for future work to reveal the cellular mechanisms underlying the NCLs.

## Background

### Neuronal ceroid lipofuscinosis

The neuronal ceroid lipofuscinoses (NCLs), commonly known as Batten disease, are devastating forms of neurodegeneration that affect the global population [1]. Mutations have been documented in 13 genetically distinct genes (*CLN1-CLN8*, *CLN10-CLN14*), each of which causes a specific subtype of the disease (e.g., mutations in *CLN3* cause CLN3 disease) [2, 3]. While the NCLs affect all ages and ethnicities, the disease is recognized as the most common form of childhood neurodegeneration [1]. Clinical manifestations of the NCLs include seizures, vision loss, reduced mental capacity, decline in motor function, and a shortened lifespan [4]. At the cellular level, ceroid lipofuscin accumulates in neurons, as well as other cell types outside of the central nervous system, due to aberrant lysosomal function [5]. Unfortunately, there is currently no cure for the NCLs, in large part due to our poor understanding of the proteins associated with the disease. As a result, other than *Brineura*, which is an enzyme replacement therapy specific for only one subtype of the disease (CLN2 disease), there are currently no effective treatments to prevent or delay the NCLs [6].

### *Dictyostelium* as a model organism for NCL research

Many animal and mammalian cell models have been used to study the pathological mechanisms underlying the NCLs [7, 8]. However, these systems present some limitations, such as the costs associated with maintaining small experimental animals (e.g., mice) and the limited number of phenotypes that can be screened in immortalized or patient-derived human cells. Not surprisingly, model organisms such as yeast, worm, fruit fly, and the social amoeba *Dictyostelium discoideum* have been used to study the roles of NCL proteins within the cell and in the context of a whole organism [8]. Collectively, this work has provided valuable insight into the localizations and functions of NCL proteins in human cells.

*Dictyostelium discoideum*, hereafter referred to simply as *Dictyostelium*, has emerged as an exceptional model system for not only studying the NCLs, but also a variety of other human neurological disorders [9, 10]. In addition to NCL proteins, *Dictyostelium* has been used to study the functions of proteins associated with Alzheimer’s disease, Parkinson’s disease, Huntington’s disease, prion diseases, epilepsy, and lissencephaly [11–16]. The 34 Mb haploid genome of *Dictyostelium* encodes an estimated 12,500 proteins, which includes homologs of 11 of the 13 human NCL proteins [9]. The life cycle of *Dictyostelium*, which has been studied for over 85 years, is comprised of unicellular and multicellular phases that allow a diversity of fundamental cellular and developmental processes to be examined in rich detail [17]. During the unicellular growth phase, cells feed on a food source such as bacteria, and proliferate via mitosis. Once the food source is depleted, cells enter a 24-h multicellular developmental programme that concludes with the formation of a fruiting body. The fruiting body is composed a slender stalk that supports a mass of viable spores that germinate and restart the life cycle once a food source becomes available. In humans, diseases that affect the nervous system share common cellular features such as the accumulation of autophagic vacuoles and mitochondrial dysfunction. These processes, along with other fundamental cellular and developmental processes including cell proliferation, chemotaxis, and differentiation, can be thoroughly studied in *Dictyostelium* using robust and high-throughput assays [17, 18]. In addition, the metazoan-like behaviour of *Dictyostelium* cells ensures a high probability that findings from the organism can be successfully translated to human cells. However, as with any model organism, there are some limitations to consider. For example, *Dictyostelium* lacks a central nervous system and has a limited number of cell types, which may prevent the translation of findings to specific tissues or organs in mammalian systems. Despite these caveats, *Dictyostelium* provides exceptional opportunities to test hypotheses related to NCL protein function in a simple eukaryotic organism that can complement and possibly inform research in mammalian systems.

### The molecular networking of NCL proteins

Since mutations in NCL proteins cause the accumulation of ceroid lipofuscin within cells, and result in broadly similar clinical manifestations between the different subtypes, the proteins are thought to have shared functions, regulate similar processes, and/or participate in shared or converging pathways [19]. In support of this hypothesis, model systems used to study the NCLs show common phenotypes [8]. At the cellular level, NCL proteins display similar localizations, have common binding partners, and regulate the expression and activities of one another. Understandably, much of this work was performed in mammalian models. However, as noted above, valuable insights have also been obtained from *Dictyostelium*. For example, many of the NCL protein homologs in *Dictyostelium* localize to the macropinocytic pathway, which is responsible for bringing fluid-phase material into the cell [20]. In addition, cell lines lacking NCL proteins display similar phenotypes [10]. This work, coupled with observations in mammalian models of NCL, suggests that NCL proteins are connected at the molecular level. These findings, and others, will be described in the sections below to help shed light on the networking of NCL proteins within the eukaryotic cell.

## Main text

### NCL proteins localize to intracellular trafficking pathways

#### Mammals

**Fig. 1:**
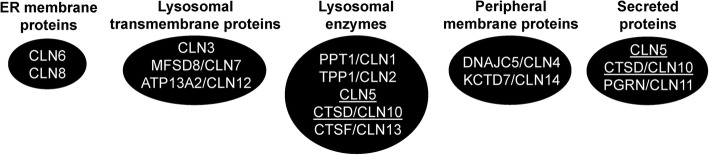
The localizations of NCL proteins in mammalian cells. Note that proteins present in more than one group are underlined

Early work in mammalian cells revealed similar subcellular localizations of some of the NCL proteins suggesting that a common cellular process may be disrupted in the NCLs [21]. Concerted research efforts have now placed the NCL proteins within intracellular trafficking pathways (e.g., endoplasmic reticulum, Golgi complex, lysosomes, vesicles), which is consistent with the lysosome dysfunction and ceroid lipofuscin accumulation observed in many cellular models of NCL (Fig. 1) [22]. Palmitoyl protein thioesterase 1 (PPT1)/CLN1, tripeptidyl peptidase 1 (TPP1)/CLN2, ceroid lipofuscinosis neuronal 5 (CLN5), cathepsin D (CTSD)/CLN10, and CTSF/CLN13 localize to the lysosome and function as soluble enzymes [23–28]. CLN5 and CTSD/CLN10 also localize extracellularly [25, 29]. CLN3 localizes to the Golgi complex and lysosomal membrane [30, 31]. Major facilitator superfamily domain-containing protein 8 (MFSD8)/CLN7 and ATPase cation-transporting 13A2 (ATP13A2)/CLN12 localize to the lysosomal membrane, while CLN6 and CLN8 are endoplasmic reticulum (ER) membrane proteins [32–35]. DnaJ homolog subfamily C member 5 (DNAJC5)/CLN4 and potassium channel tetramerization domain-containing protein 7 (KCTD7)/CLN14 are peripheral membrane proteins, while progranulin (PGRN)/CLN11 is secreted [36–38].

#### Dictyostelium

Nine of the thirteen human NCL proteins have clear homologs in *Dictyostelium*, which includes homologs of PPT1/CLN1 (Ppt1), TPP1/CLN2 (see next sentence), CLN3 (Cln3), DNAJC5/CLN4 (Dnajc5), CLN5 (Cln5), MFSD8/CLN7 (Mfsd8), CTSD/CLN10 (CtsD), PGRN/CLN11 (Grn), and ATP13A2/CLN12 (Kil2) [9]. Six proteins in *Dictyostelium* share sequence similarity with human TPP1/CLN2 (Tpp1A, Tpp1B, Tpp1C, Tpp1D, Tpp1E, Tpp1F) [39]. However, of the six proteins, Tpp1A and Tpp1B are closest in size to human TPP1/CLN2 (600 and 598 amino acids, respectively, compared to 563 amino acids) and have the highest sequence similarity. The expression of *tpp1B* is markedly higher than the other *tpp1* genes during growth and the early stages of multicellular development [8]. *tpp1F* is also highly expressed during growth relative to *tpp1A*, *tpp1C*, *tpp1D*, and *tpp1E*. Upon starvation, the expression of *tpp1B* and *tpp1F* decrease dramatically reaching their lowest levels once the multicellular aggregate has formed and remain low during the mid-to-late stages of development. In contrast, the expression of *tpp1A* increases dramatically upon starvation, and remains high during the mid-to-late stages of multicellular development. During this time, the expression levels of *tpp1B*, *tpp1C*, *tpp1D*, *tpp1E*, and *tpp1F* are comparatively lower. Together, these expression profiles suggest that Tpp1B and Tpp1F may be the dominant Tpp1 proteins during growth, while Tpp1A may be the dominant Tpp1 protein during development. Since one TPP1/CLN2 protein has been identified in mammalian cells, it will be important for future work to reveal the true homolog of human TPP1/CLN2 in *Dictyostelium*. In addition, the *Dictyostelium* genome encodes several proteins that share similarity with human CTSF/CLN13 and KCTD7/CLN14 [8]. Specific proteins have been identified as the likely homologs of human CTSF/CLN13 and KCTD7/CLN14, however they have not been experimentally validated. Finally, the *Dictyostelium* genome does not encode homologs of human CLN6 or CLN8 [9]. Interestingly, these are the only ER-resident proteins among the family of NCL proteins. Current knowledge indicates that the *Dictyostelium* ER shares several functions with the ER in mammalian cells including calcium storage, lipid biosynthesis, and the unfolded protein response [40]. Furthermore, *Dictyostelium* is the only microorganism known to encode homologs of human calnexin and calreticulin [41]. So, it is somewhat surprising that *Dictyostelium* does not contain homologs of CLN6 and CLN8. Their absence in *Dictyostelium* could indicate a specialized role for these proteins in the ER of mammalian cells that is either non-existent or dispensable in *Dictyostelium*. These observations suggest that future work on CLN6 and CLN8 in mammalian cells may consider focusing on specialized or highly evolved ER-dependent processes that are not present in evolutionarily lower eukaryotes; processes that may or may not be cell type-dependent.

**Fig. 2:**
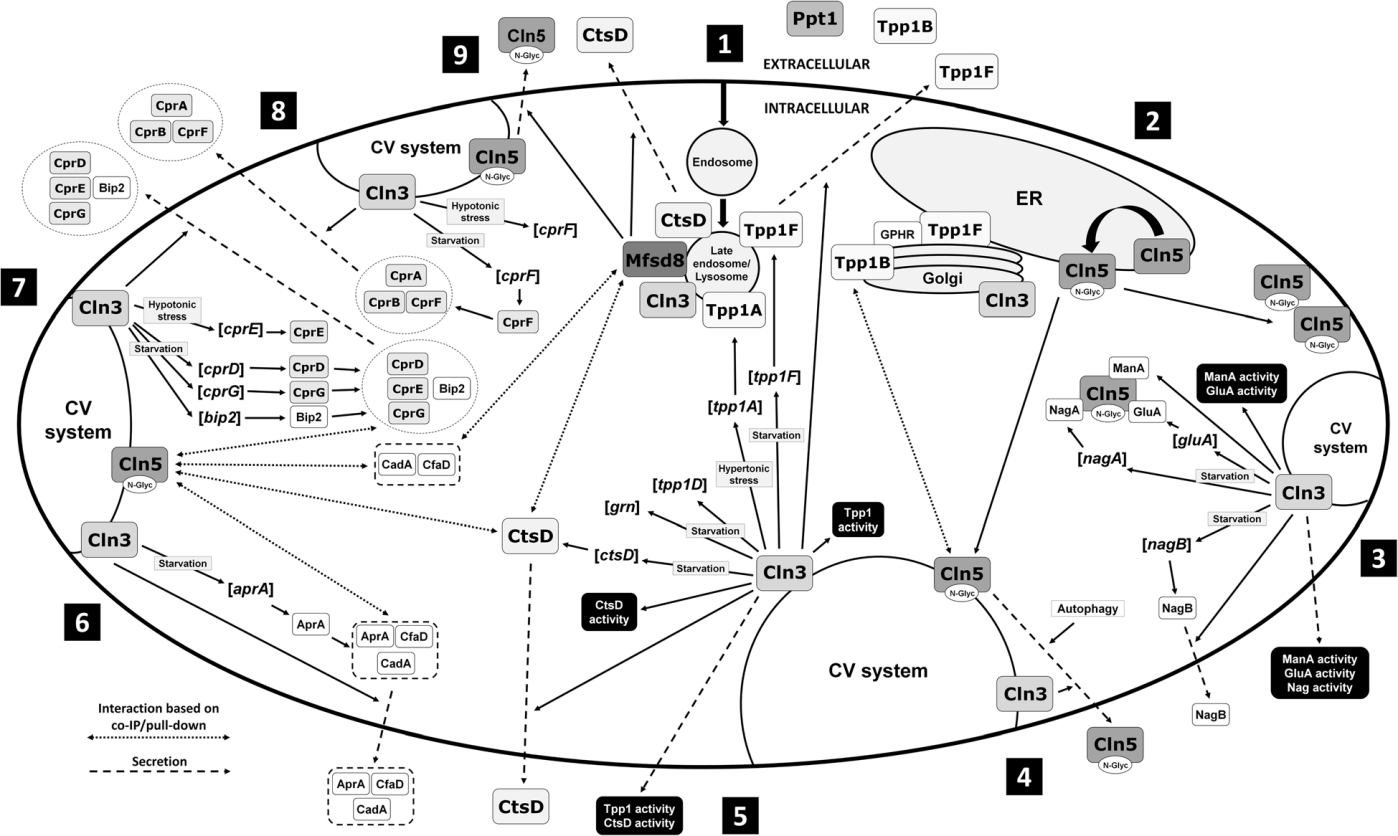
The networking of NCL proteins in *Dictyostelium*. (1) Material is taken up by the cell and incorporated into an endosome, which matures into a lysosome. Tpp1A, Tpp1F, Cln3, Mfsd8, and CtsD localize to the late endosome/lysosome. Tpp1F also localizes extracellularly as does Ppt1 and Tpp1B. (2) Tpp1B localizes to the Golgi complex and binds the Golgi pH regulator (GPHR) (in addition to extracellularly, see #1). Tpp1F localizes to the Golgi complex and endoplasmic reticulum (ER) (in addition to late endosome/lysosome and extracellularly, see #1) and binds the GPHR. Cln3 localizes to the Golgi complex (in addition to the late endosome/lysosome, see #1). Cln5 is glycosylated in the ER and then trafficked to the cell cortex and contractile vacuole (CV) system. (3) Cln3 localizes to the CV system (in addition to the late endosome/lysosome and Golgi complex, see #1 and #2). During starvation, loss of *cln3* alters the intracellular activity of alpha-mannosidase (ManA), the expression of beta-glucosidase (*gluA*), the intracellular activity of GluA, and the expression of N-acetylglucosaminidase (*nagA*). Cln5 interacts with ManA, GluA, and NagA. Loss of *cln3* alters the expression of *nagB* and the secretion of NagB during starvation. Finally, loss of *cln3* alters the extracellular activities of ManA, GluA, and Nag during starvation. (4) Cln5 is secreted during starvation. Secretion of Cln5 is regulated by autophagic mechanisms (i.e., autophagy inhibition decreases secretion) and Cln3 (i.e., *cln3*-deficiency alters secretion). Inside the cell, Cln5 interacts with Tpp1B. (5) Loss of *cln3* alters the intracellular and extracellular activity of Tpp1 during starvation. *cln3*-deficiency alters the expression of *tpp1F* and the secretion of Tpp1F during starvation. Loss of *cln3* increases the expression of *tpp1A* during hypertonic stress and alters the expression of *tpp1D* and *grn* during starvation. *cln3*-deficiency alters the expression of *ctsD*, the intracellular and extracellular activity of CtsD, and the secretion of CtsD during starvation. (6) Loss of *cln3* alters the expression of *aprA* and the intracellular amount of AprA during starvation. *cln3*-deficiency alters the secretion of AprA during growth and starvation. Loss of *cln3* alters the secretion of CfaD during growth and the amount of CadA in conditioned starvation buffer. Cln5 interacts with AprA, CfaD, CadA, and CtsD. (7) Loss of *cln3* alters the expression of *cprD, cprG,* and *bip2* (luminal-binding protein 2, DDB0233663) during starvation. *cln3*-deficiency increases the expression of *cprE* during hypotonic stress. Loss of *cln3* alters the secretion of CprD, CprE, CprG, and Bip2 during starvation. Cln5 interacts with CprD, CprE, CprG, and Bip2. (8) Loss of *cln3* alters the expression of *cprF* during hypotonic stress and starvation. *cln3*-deficiency alters the secretion of CprA, CprB, and CprF during starvation. (9) Like Cln5, Mfsd8 interacts with CtsD, CadA, and CfaD (see #6). Like *cln3*^*−*^ cells (see #4 and #5), loss of *mfsd8* alters the secretion of Cln5 and CtsD during starvation

A proteomics-based analysis revealed that 8 of the 9 clear NCL protein homologs in *Dictyostelium* localize to the macropinocytic pathway, which is responsible for bringing fluid-phase material into the cell [8, 20]. In addition, some of the proteins that share similarity with human CTSF/CLN13 and KCTD7/CLN14 also localize to this pathway [8, 20]. More directed studies have also provided support for the localization of NCL proteins to intracellular trafficking pathways in *Dictyostelium*. For example, the *Dictyostelium* homologs of human TPP1/CLN2 (Tpp1A, Tpp1F), CLN3 (Cln3), MFSD8/CLN7 (Mfsd8), and CTSD/CLN10 (CtsD) all localize to the late endosome/lysosome suggesting they play a role in the digestion of ingested material [39, 42–46] (Fig. 2). In addition to the late endosome/lysosome, Tpp1F also localizes to the ER and Golgi complex indicating that the protein is likely synthesized in the ER, processed in the Golgi complex, and then trafficked to the lysosome [39]. Like Tpp1F, Tpp1B and Cln3 also localize to the Golgi complex [39, 47].

Cln5 is glycosylated in *Dictyostelium*, and not surprisingly, the protein is detected in the ER [26, 48] (Fig. 2). Cln5 also localizes to the cell cortex and contractile vacuole (CV) system [26, 48]. During the early stages of multicellular development, Cln5 is detected extracellularly, which aligns with observations in mammalian cells [25, 26, 48–50]. The secretion of Cln5 in *Dictyostelium* appears to be regulated by autophagic mechanisms and Cln3, which also localizes to the CV system [43, 45, 47, 48]. While the main function of the CV system in *Dictyostelium* is osmoregulation, the organelle has also been linked to unconventional protein secretion [51]. Interestingly, experimental evidence suggests that regulated intracellular transport might occur between the CV and endo-lysosomal systems, which provides further support for the localization of NCL proteins to intracellular trafficking pathways [52–54]. Consistent with the localization of GFP-Cln3 to the CV system in *Dictyostelium*, hypertonic stress dramatically increases the expression of human myc-tagged CLN3 mRNA and protein in baby hamster kidney cells [55]. Finally, like Cln5, Ppt1, Tpp1B, Tpp1F, and CtsD are also secreted during *Dictyostelium* development suggesting that NCL proteins may play an important role outside the cell [42, 47, 56].

Together, these findings show that homologs of NCL proteins in *Dictyostelium* may play an important role in regulating intracellular trafficking mechanisms. These findings are consistent with observations in mammalian systems and support the notion that aberrant processing of intracellular material is a key component of NCL pathology.

### Protein interactomes shed light on the interactions between NCL proteins

#### Mammals

Modern omics technologies have the potential to provide significant new insight into protein interactomes, potential biomarkers, and biological pathways affected by the NCLs [57]. For example, proteomics-based analyses in SH-SY5Y human neuroblastoma cells revealed that PPT1/CLN1, CLN3, and CLN5 interact with 23, 58, and 31 proteins, respectively [58, 59]. A comparison of the three interactomes revealed several proteins that may serve important functions in the NCL-causing pathway (Table 1). PPT1/CLN1 and CLN3 share four common interactors (ATP1A1, DBH, SLC25A1, SLC25A13). Three of those proteins (DBH, SLC25A1, SLC25A13) also interact with CLN5. DBH (dopamine β-hydroxylase) is a copper-containing oxygenase that catalyzes the conversion of dopamine to norepinephrine [60]. Not surprisingly, altered dopamine signalling has been linked to the NCLs, as well as other neurological disorders including Alzheimer’s disease, Parkinson’s disease, and schizophrenia [61–65]. Mutations in *SLC25A1* are associated with impaired neuromuscular transmission [66]. *SLC25A1* encodes a mitochondrial tricarboxylate transport protein that regulates the exchange of mitochondrial citrate for cytosolic malate and contributes to fatty acid and sterol biosynthesis [67]. Intriguingly, mutations in NCL genes alter lipid homeostasis [68–70]. Finally, SLC25A13 is a calcium-binding carrier protein that catalyzes the exchange of cytoplasmic glutamate with aspartate across the inner mitochondrial membrane [71, 72]. Mutations in *SLC25A13* cause citrullinemia, an autosomal recessive disease characterized by elevated levels of ammonia in the blood, which leads to neuropsychiatric symptoms such as abnormal behaviour, memory loss, seizures, and even death [73]. Importantly, mitochondrial dysfunction has been reported in the NCLs and there is evidence supporting altered ammonia homeostasis in at least one subtype of the disease (e.g., CLN3 disease) [74–76]. Together, these findings suggest that further investigation into the involvement of DBH, SLC25A1, and SLC25A13 in NCL pathology is warranted.

**Table 1:** List of PPT1/CLN1, CLN3, and CLN5 common interactors in human cells

Protein	PPT1/CLN1	CLN3	CLN5
ARF4	No	**Yes**	**Yes**
ATP1A1	**Yes**	**Yes**	No
ATP2A2	No	**Yes**	**Yes**
CALU	No	**Yes**	**Yes**
CDS2	No	**Yes**	**Yes**
**DBH**	**Yes**	**Yes**	**Yes**
PHGDH	No	**Yes**	**Yes**
RCN2	No	**Yes**	**Yes**
RPN1	No	**Yes**	**Yes**
SEC61A1	No	**Yes**	**Yes**
SFXN3	No	**Yes**	**Yes**
**SLC25A1**	**Yes**	**Yes**	**Yes**
SLC25A11	No	**Yes**	**Yes**
**SLC25A13**	**Yes**	**Yes**	**Yes**
SLC25A22	No	**Yes**	**Yes**
SLC25A4	No	**Yes**	**Yes**
SLC25A5	No	**Yes**	**Yes**
SLC25A6	No	**Yes**	**Yes**
XPO1	No	**Yes**	**Yes**

Proteomics-based analyses have also provided evidence that NCL proteins interact with each other (Table 2). For example, PPT1/CLN1 was shown to interact with CTSD/CLN10 in SH-SY5Y human neuroblastoma cells, possibly due to their common function as lysosomal enzymes [23, 27, 59]. In addition, co-immunoprecipitation and in vitro binding assays revealed that CLN5 polypeptides interact directly with TPP1/CLN2 and CLN3 in COS-1 cells [77]. These interactions were later confirmed in a study that also identified PPT1/CLN1, CLN6, and CLN8 as CLN5-interactors in mouse brain extract, COS-1 cells, and HeLa cells [78]. These findings are significant since they highlight a conserved interaction between CLN5 and these NCL proteins across multiple models. Interestingly, work has shown that the intracellular trafficking of CLN5 in HeLa cells is regulated by PPT1/CLN1 and may involve F1-ATPase, a common interactor of PPT1/CLN1 and CLN5 [78]. The association of CLN5 with PPT1/CLN1 and TPP1/CLN2 could reflect their common function as lysosomal enzymes, albeit with distinctly different biological activities [23, 24, 26] (Fig. 1). The interaction between CLN3 and CLN5 could be due to their common link to Rab7 and the retromer complex, while the binding of CLN5 to CLN6 and CLN8 could indicate that these proteins play a role in processing CLN5 to its mature form in the ER [84–86]. Finally, CTSD/CLN10 has been shown to interact with PGRN/CLN11 in HEK293T cells, which could possibly be explained by the observations that both proteins are secreted [29, 38, 79, 80] (Table 2, Fig. 1). Together, these results suggest that the networking of NCL proteins within the cell may involve direct interactions with each other.

**Table 2:** Interactions between NCL proteins in mammals

Protein 1	Protein 2	Effect	Sample	Reference
PPT1/CLN1	CTSD/CLN10	Interaction	SH-SY5Y human neuroblastoma cells	Scifo et al., 2013 [59]
CLN5	TPP1/CLN2	Interaction	COS-1 cells	Vesa et al., 2002 [77]
	CLN3			
CLN5	PPT1/CLN1	Interaction	Mouse brain extract COS-1 cells (author note: place on a separate line under "Mouse brain extract") HeLa cells (author note: place on a separate line under "COS-1 cells")	Lyly et al., 2009 [78]
	TPP1/CLN2			
	CLN3			
	CLN6			
	CLN8			
CTSD/CLN10	PGRN/CLN11	Interaction	HEK293T cells	Zhou et al., 2017; Valdez et al., 2017 [79, 80]
PPT1/CLN1	DNAJC5/CLN4	PPT1/CLN1 depalmitoylates DNAJC5/CLN4	PPT1/CLN1 from HEK293T cell media depalmitoylated DNAJC5/CLN4 isolated from mouse brain	Henderson et al., 2016 [81]
PPT1/CLN1	CLN5	PPT1/CLN1 regulates the trafficking of CLN5	HeLa cells	Lyly et al., 2009 [78]
CLN3	CTSD/CLN10	Co-localization under hypertonic conditions	Baby hamster kidney cells	Cárcel-Trullols et al., 2017 [82]
Increased expression of *CLN3* under hypertonic conditions	CTSD/CLN10	Decreased activity of CTSD/CLN10	Baby hamster kidney cells	Cárcel-Trullols et al., 2017 [82]
CLN5	CTSD/CLN10	CLN5 regulates the processing of CTSD/CLN10	CLN5 (N320S) expressed in HeLa cells reduced the processing of CTSD/CLN10 to its enzymatically active form	Qureshi et al., 2018 [83]
PGRN/CLN11	CTSD/CLN10	PGRN/CLN11 increases the activity of CTSD/CLN10	Human PGRN and CTSD/CLN10 from bovine spleen	Valdez et al., 2017 [80]

#### Dictyostelium

Recent work in *Dictyostelium* reported the Cln5 and Mfsd8 protein interactomes [26, 46]. In addition to identifying the interactors of each protein, these studies also provided insight into the molecular interactions between NCL protein homologs in *Dictyostelium*. For example, this work showed that Cln5 interacts with Tpp1B and CtsD [26] (Fig. 2). In mammalian cells, CLN5 interacts with TPP1/CLN2, and increased TPP1/CLN2 activity has been observed in fibroblasts from a CLN5 disease patient [77, 78] (Table 2, Table 3). In addition to Cln5, CtsD was also reported to bind to Mfsd8 in *Dictyostelium*, thus establishing a molecular connection between Cln5, Mfsd8, and CtsD in this model organism [46] (Fig. 2). The binding of MFSD8/CLN7 to CTSD/CLN10 has not been reported in mammalian cells but results from *Dictyostelium* suggest they may interact with each other.

**Table 3:** Loss of NCL protein function in mammals and the effects on the expression, amounts, and activities of NCL genes/proteins

**Loss of PPT1/CLN1 function**	**Sample**	**Affected gene/protein**	**Reference**
*Ppt1/Cln1*^*−/−*^	Brain tissues and cells from a *Ppt1/Cln1*^*−/−*^ mouse	Increased expression of *CtsD/Cln10*	Chandra et al., 2015 [87]
*Ppt1/Cln1*^*−/−*^	Brain tissues and cells from a *Ppt1/Cln1*^*−/−*^ mouse	Reduced maturation of CTSD/CLN10 to its enzymatically active form	Chandra et al., 2015 [87]
**Loss of TPP1/CLN2 function**	**Sample**	**Affected gene/protein**	**Reference**
*Tpp1/Cln2*^*−/−*^	Brain tissues from a *Tpp1/Cln2*^*−/−*^ mouse	Increased expression of *CtsD/Cln10*	Domowicz et al., 2019 [88]
*Tpp1/Cln2*^*−/−*^	Brain tissues from a *Tpp1/Cln2*^*−/−*^ mouse	Increased expression of *Pgrn/Cln11*	Domowicz et al., 2019 [88]
**Loss of CLN3 function**	**Sample**	**Affected gene/protein**	**Reference**
*CLN3* (mutated)	CLN3 disease patient fibroblasts	Reduced amount of PPT1/CLN1 in lysosomes, which correlated with reduced enzymatic activity	Appu et al., 2019 [89]
*Cln3*^*Δex7/8*^	Mouse brain cortex from a *Cln3*^*Δex7/8*^ mouse	Reduced amount of PPT1/CLN1 in lysosomes, which correlated with reduced enzymatic activity	Appu et al., 2019 [89]
*Cln3*^*Δex7/8*^	Cerebellar cells from a *Cln3*^*Δex7/8*^ mouse	Increased amount of PPT1/CLN1 in lysosomes, which correlated with increased enzymatic activity	Schmidtke et al., 2019 [90]
*Cln3*^*Δex7/8*^	Cerebellar cells from a *Cln3*^*Δex7/8*^ mouse	Increased amount of TPP1/CLN2 in lysosomes	Schmidtke et al., 2019 [90]
*Cln3*^*Δex7/8*^	Cerebellar cells from a *Cln3*^*Δex7/8*^ mouse	Decreased amount of CLN5 in lysosomes	Schmidtke et al., 2019 [90]
*Cln3*^*Δex7/8*^	Cerebellar cells from a *Cln3*^*Δex7/8*^ mouse	Decreased amount of CTSD/CLN10 in lysosomes	Schmidtke et al., 2019 [90]
**Loss of DNAJC5/CLN4 function**	**Sample**	**Affected gene/protein**	**Reference**
*DNAJC5/CLN4* (mutated)	CLN4 disease patient brain	Increased amount of PPT1/CLN1	Henderson et al., 2016 [81]
*DNAJC5/CLN4* (mutated)	CLN4 disease patient brain	Reduced enzymatic activity of PPT1/CLN1, which resulted in its mis-localization	Henderson et al., 2016 [81]
**Loss of CLN5 function**	**Sample**	**Affected gene/protein**	**Reference**
*CLN5* (mutated)	CLN5 disease patient fibroblasts	Increased activity of TPP1/CLN2	Vesa et al., 2002 [77]
**Loss of MFSD8/CLN7 function**	**Sample**	**Affected gene/protein**	**Reference**
*Mfsd8/Cln7*^*−/−*^	Embryonic fibroblasts from a *Mfsd8/Cln7*^*−/−*^ mouse	Reduced amount of CLN5 in lysosomes	Danyukova et al., 2018 [91]
**Loss of PGRN/CLN11 function**	**Sample**	**Affected gene/protein**	**Reference**
*PGRN/CLN11* (heterozygous mutation)	FTD patient fibroblasts	Reduced activity of CTSD/CLN10	Ward et al., 2017 [92]
*PGRN/CLN11* (heterozygous mutation)	FTD patient iPSC-derived cortical neurons	Reduced activity of CTSD/CLN10	Valdez et al., 2017 [80]
*PGRN/CLN11*^*−/−*^	Liver, spleen, and brain samples from a *PGRN/CLN11*^*−/−*^mouse	Reduced activity of CTSD/CLN10	Zhou et al., 2017 [79]
**Loss of ATP13A2/CLN12 function**	**Sample**	**Affected gene/protein**	**Reference**
*ATP13A2/CLN12* knockdown	SH-SY5Y human neuroblastoma cells	Reduced amount and activity of CTSD/CLN10	Matsui et al., 2013 [93]

As discussed previously, CLN5 interacts with CLN3 in mammalian cells [77, 78]. Several pieces of evidence also support a connection between Cln3 and Cln5 in *Dictyostelium*. During starvation, *cln3*-deficiency alters the expression of N-acetylglucosaminidase (*nagA*) and beta-glucosidase (*gluA*) [94] (Fig. 2). *cln3*-deficiency also alters the intracellular and extracellular activity of alpha-mannosidase (ManA) and beta-glucosidase (GluA) during starvation, as well as the extracellular activity of N-acetylglucosaminidase (Nag) [94]. Intriguingly, Cln5 interacts with ManA, GluA, and NagA, thus establishing a network involving Cln3, Cln5, and these lysosomal enzymes in *Dictyostelium* [26]. In support of these findings, recent work reported abnormal enzyme amounts in lysosomal fractions from cerebellar cells derived from a *Cln3*^*Δex7/8*^ mouse [90] (Table 3).

In *Dictyostelium*, *cln3*-deficiency increases cell proliferation during growth and reduces cell adhesion during the early stages of multicellular development [43, 45]. To help explain these phenotypes, previous work showed that *cln3*-deficiency alters the secretion of autocrine proliferation repressor A (AprA) and counting factor-associated protein D (CfaD) during growth, the expression and secretion of AprA during starvation, and the extracellular amount of calcium-dependent cell adhesion protein A (CadA) during starvation [43, 45, 94] (Fig. 2). The connection between Cln3 and Cln5 in *Dictyostelium* is strengthened by the finding that AprA, CfaD, and CadA have all been identified as Cln5-interactors [26]. As discussed earlier, CtsD was identified in both the Cln5 and Mfsd8 interactomes [26, 46]. Not surprisingly, CfaD and CadA were also identified as Mfsd8-interactors further supporting the molecular connection between Cln5 and Mfsd8 in *Dictyostelium* [46].

The *Dictyostelium* genome encodes several cysteine proteinases (Cpr) that share sequence similarity with human CTSF/CLN13. Three of these proteins, CprD, CprE, and CprG, were identified in the Cln5 interactome [26] (Fig. 2). In keeping with the connection between Cln3 and Cln5 in *Dictyostelium*, loss of *cln3* alters the expression of *cprD, cprF*, and *cprG* during starvation and increases the expression of *cprE* and *cprF* during hypotonic stress [94, 95]. *cln3*-deficiency also alters the secretion of CprA, CprB, CprD, CprE, CprF, and CprG [47]. These results suggest that future research in mammalian models of NCL may consider examining the molecular interactions between CLN3, CLN5, and CTSF/CLN13.

In *Dictyostelium*, Cln5 interacts with luminal-binding protein 2 (Bip2, DDB0233663), whose expression and secretion are affected by the loss of *cln3* during starvation [26, 47, 94] (Fig. 2). Bip2 is massively over-secreted by *cln3*^*−*^ cells compared to WT cells during starvation and is one of the most significantly affected proteins [47]. Bip2 is the *Dictyostelium* homolog of human glucose-regulated protein 78 (GRP78)/BiP, which plays an important role in cancer progression and neurodegeneration [96]. Intriguingly, CLN3 interacts with GRP78/BiP in COS-1 cells [97]. Together, this research has set the stage for future work in mammalian models of NCL to explore the molecular connection between CLN3 and CLN5.

Finally, as discussed above, DBH, SLC25A1, and SLC25A13 all interact with PPT1/CLN1, CLN3, and CLN5 in SH-SY5Y human neuroblastoma cells [58, 59]. While the *Dictyostelium* genome does not encode a homolog of DBH, it does encode several homologs of human SLC25A1 and SLC25A13 (www.dictybase.org). Homologs of SLC25A1 and SLC25A13 have been identified as Mfsd8-interactors in *Dictyostelium* (mitochondrial substrate carrier family protein, McfG, McfN, McfT, McfZ) [46]. In addition, several Mcf proteins are differentially expressed in *cln3*^*−*^ cells compared to WT during starvation (McfC, McfF, McfG, McfM, McfR) [94]. Therefore, *Dictyostelium* presents an excellent system to dissect the roles of SLC25A1 and SLC25A13 in NCL biologys.

### Loss or mutation of individual NCL genes affects other NCL genes/proteins

Several studies in *Dictyostelium* and mammalian models of NCL have shown that loss or mutation of a single NCL gene can affect the expression, amounts, and/or enzymatic activities of other NCL genes/proteins. These studies will be described in the sections below to highlight the links between individual NCL genes and other NCL genes/proteins.

#### Loss of *PPT1/CLN1* affects CTSD/CLN10

Brain tissues and cells from *Ppt1/Cln1*^*−/−*^ mice display increased expression of *CtsD/Cln10* [87] (Table 3). Loss of PPT1/CLN1 function in mice also disrupts the maturation of CTSD/CLN10 to its enzymatically active form, which leads to an accumulation of undegraded cargo in the lysosome [87]. These observations are further supported by the finding that CTSD/CLN10 interacts with PPT1/CLN1 in SH-SY5Y human neuroblastoma cells, which may reflect their common function as lysosomal enzymes [23, 27, 59] (Fig. 1, Table 2). Together, these results suggest a link between PPT1/CLN1 and CTSD/CLN10.

#### Loss of *CLN3* affects PPT1/CLN1, TPP1/CLN2, CLN5, CTSD/CLN10, and PGRN/CLN11

Lysosomes isolated from the cortex of a *Cln3*^*Δex7/8*^ mouse brain, as well as fibroblasts from CLN3 disease patients, progressively accumulate autofluorescent ceroid and contain significantly reduced levels of PPT1/CLN1, which corelate with reduced PPT1/CLN1 enzymatic activity [89] (Table 3). The significance of these observations is strengthened by their detection in different model systems. Interestingly, a study published in parallel reported an increased amount and activity of PPT1/CLN1 in lysosomes from cerebellar cells isolated from the same mouse model [90]. While the explanation for this discrepancy is not immediately clear, it may reflect a cell type dependent effect of mutated *Cln3* on the expression and activity of PPT1/CLN1 in mice (e.g., cortex vs. cerebellum). Along with PPT1/CLN1, Schmidtke et al. [90] also reported an increased amount of TPP1/CLN2 in lysosomal fractions from cerebellar cells derived from a *Cln3*^*Δex7/8*^ mouse. Results from *Dictyostelium* are consistent with these observations since loss of *cln3* alters intracellular and extracellular Tpp1 activity during starvation [94] (Fig. 2). *cln3*-deficiency also alters the expression of *tpp1A* during hypertonic stress, the expression of *tpp1D* during starvation, and the expression and secretion of Tpp1F during starvation [47, 94, 95]. Therefore, data from *Dictyostelium* and mammals support a molecular interaction between PPT1/CLN1, TPP1/CLN2, and CLN3, which may be explained the proposed role of CLN3 in the trafficking of lysosomal enzymes [85, 90].

Lysosomal fractions from cerebellar cells derived from a *Cln3*^*Δex7/8*^ mouse also display decreased amounts of CLN5 and CTSD/CLN10, as well as other lysosomal enzymes such as hexosaminidase A, hexosaminidase B, and alpha-mannosidase [90] (Table 3). In baby hamster kidney cells, hypertonic stress increases the expression of human myc-tagged CLN3 mRNA and protein [55]. Under these conditions, human myc-tagged CLN3 co-localizes with CTSD/CLN10 and decreases the activity of CTSD/CLN10 but has no effect on the amount of CTSD/CLN10 protein [82] (Table 2). In *Dictyostelium*, loss of *cln3* alters the expression of *ctsD*, the intracellular and extracellular activity of CtsD, and the secretion of Cln5 [48, 94] (Fig. 2). *cln3*-deficiency also alters the expression of the *Dictyostelium* homologs of hexosaminidase A and B (*nagA* and *nagB*) [94]. Intriguingly, Cln5 interacts with NagA and alpha-mannosidase, which are affected by the loss of CLN3 function in mice [26, 90]. Together, these results establish links between CLN3, CLN5, and CTSD/CLN10. Like PPT1/CLN1 and TPP1/CLN2, the connection between these proteins may be explained by the proposed role of CLN3 in the trafficking of lysosomal enzymes [85, 90]. Finally, loss of *cln3* in *Dictyostelium* affects the expression of *grn* during starvation, which could be linked to the role of Cln3 in protein secretion (i.e., PGRN/CLN11 is secreted in mammalian cells) [47]. Importantly, this finding suggests there may be a molecular interaction between CLN3 and PGRN/CLN11 in mammalian cells.

#### Loss of *DNAJC5/CLN4* affects PPT1/CLN1

In brain tissue obtained from CLN4 disease patients, mutations in *DNAJC5/CLN4* increase the amount of PPT1/CLN1 but reduce its enzymatic activity causing it to mis-localize [81] (Table 2, Table 3). Consistent with these findings, PPT1/CLN1 isolated from HEK293T cell media was shown to de-palmitoylate DNAJC5/CLN4 isolated from mouse brain [81]. While the link between the two proteins is not entirely clear, the data suggest that PPT1/CLN1 may regulate DNAJC5/CLN4 function through de-palmitoylation. Also, since DNAJC5/CLN4 has been linked to the trafficking of synaptic vesicles, it is possible that DNAJC5/CLN4 may regulate the trafficking of PPT1/CLN1 [36]. While additional work is required to clarify the link between DNAJC5/CLN4 and PPT1/CLN1, these observations suggest a molecular association may exist between the two proteins.

#### Loss of *CLN5* affects TPP1/CLN2 and CTSD/CLN10

CLN5 disease patient fibroblasts display increased TPP1/CLN2 activity [77] (Table 3). In support of these findings, CLN5 has been shown to interact with TPP1/CLN2 in mouse brain extract, COS-1 cells, and HeLa cells (Table 2) [77, 78]. In *Dictyostelium*, Cln5 interacts with Tpp1B, which is the most highly expressed Tpp1 protein in the organism [8, 26] (Fig. 2). Recently, whole-exome sequencing of Alzheimer’s disease families revealed a missense variant in CLN5 that segregated with Alzhemier’s disease, which supports the hypothesis that there may be similarities between NCL pathology and other forms of neurodegeneration, including Alzheimer’s disease, Parkinson’s disease, and frontotemporal dementia [80, 83, 92, 98, 99]. This variant displayed glycosylation defects in CLN5 that caused it to be retained in the ER of HeLa cells, which coincided with reduced trafficking to the endo-lysosomal compartment [83]. The variant also showed reduced processing of CTSD/CLN10 and decreased levels of full-length amyloid precursor protein, thus providing evidence linking the function of CLN5 to CTSD/CLN10 (Table 2). The binding of Cln5 to CtsD in *Dictyostelium* is consistent with these observations [26] (Fig. 2). Together, these results establish links between TPP1/CLN2, CLN5, and CTSD/CLN10, which are likely reflective of their common function as lysosomal enzymes, albeit with different activities [24, 26, 27].

#### Loss of *MFSD8/CLN7* affects CLN5

A SILAC-based quantitative analysis of lysosomes from *Mfsd8/Cln7*-deficient mouse embryonic fibroblasts (MEFs) derived from a *Mfsd8/Cln7*^*−/−*^ mouse revealed that loss of *Mfsd8/Cln7* significantly depletes the amount of lysosomal CLN5, as well as other lysosomal enzymes [91] (Table 3). The depletion of CLN5 was shown to be due to increased proteolytic degradation by cysteine proteases in the lysosomes of *Mfsd8/Cln7*^*−/−*^ MEFs [91]. These findings are also supported by work in *Dictyostelium*, which showed that Mfsd8 regulates the secretion of Cln5 during the early stages of multicellular development [46] (Fig. 2). In total, these data suggest that depletion of CLN5 may contribute to the pathogenesis associated with CLN7 disease and that MFSD8/CLN7 may play an important role in the trafficking of lysosomal enzymes.

#### Is CTSD/CLN10 a common pathogenic link for all NCLs?

The findings described above suggest that loss of CTSD/CLN10 activity may be a common pathogenic link between several subtypes of NCL (i.e., links between CTSD/CLN10 and PPT1/CLN1, CLN3, and CLN5). Based on these observations, it is not surprising that loss of *TPP1/CLN2*, *PGRN/CLN11*, or *ATP13A2/CLN12* also affects the function of CTSD/CLN10. In brain tissues from a *Tpp1/Cln2*^*−/−*^ mouse model, loss of *Tpp1/Cln2* increases the expression of *CtsD/Cln10* [88] (Table 3). Loss of *Tpp1/Cln2* in this mouse model also increases the expression of *Pgrn/Cln11* suggesting there may be a molecular connection between TPP1/CLN2 and PGRN/CLN11. In liver, spleen, and brain (cerebrum, cerebellum, midbrain) lysates from *PGRN/CLN11*-deficient mice, the activity of CTSD/CLN10 is significantly reduced, without any changes in the level of CTSD/CLN10 protein [79]. These observations were independently validated in fibroblasts and induced pluripotent stem cell (iPSC)-derived cortical neurons from patients with frontotemporal dementia (FTD, carry heterozygous mutations in *PGRN/CLN11*), thus highlighting the conserved effect of PGRN/CLN11 on CTSD/CLN10 activity in multiple models [80, 92]. In support of these findings, FLAG-tagged CTSD/CLN10 was shown to interact directly with untagged PGRN/CLN11 in HEK293T cells [79] (Table 2). A parallel study confirmed the interaction between CTSD/CLN10 and PGRN/CLN11 and showed that PGRN/CLN11 of human origin can increase the activity of CTSD/CLN10 from bovine spleen [80]. Together, these findings suggest that PGRN/CLN11 activates CTSD/CLN10, which provides a possible explanation for the overlapping pathology observed in individuals with mutated *CTSD/CLN10* or *PGRN/CLN11*. Finally, in SH-SY5Y human neuroblastoma cells, knockdown of *ATP13A2/CLN12* reduces the amount and activity of CTSD/CLN10 [93] (Table 3). In summary, these findings show that at least one aspect of CTSD/CLN10 function (e.g., expression, amount, processing, activity) is linked to the expression and/or activity of six NCL genes/proteins (e.g., PPT1/CLN1, TPP1/CLN2, CLN3, CLN5, PGRN/CLN11, ATP13A2/CLN12), which constitutes half of all NCL genes/proteins.

### NCL gene expression during mammalian development and cancer

There is also evidence supporting the potential networking of NCL genes during mammalian development and cancer. During brain development in mice, *Tpp1/Cln2*, *Cln3*, and *Cln5* are spatially and temporally co-expressed [100]. Specifically, these genes are expressed in regions associated with the formation of new neurons including the germinal epithelium, ventricle regions, hippocampus, and cerebellum. In addition, the genes are temporally co-expressed in neuronal progenitor cells suggesting they may play an important role in early embryonal neurogenesis. Gene expression profiling of the cortex from *Cln1* and *Cln5* knockout mice revealed that loss of either *Ppt1/Cln1* or *Cln5* affects the expression of genes that regulate neuronal growth cone stabilization [101]. Intriguingly, the three major genes comprising this pathway (adenylate cyclase-associated protein 1, protein tyrosine phosphatase receptor type F, and protein tyrosine phosphatase 4a2) all cluster to the same locus as *Ppt1/Cln1*, further supporting a functional relationship between the gene products. In addition, immunofluorescence experiments performed on cortical neurons isolated from these mice display abnormal distribution of the cytoskeletal proteins, actin and beta-tubulin, as well as growth cone associated proteins GAP-43, synapsin, and Rab3. NCL gene expression has also been linked to various forms of cancer [102–105]. In one study, a close correlation between *PPT1/CLN1* and *TPP1/CLN2* expression was reported in the progression and metastasis associated with colorectal cancer [103]. In total, NCL gene expression during cancer and mammalian development suggests a shared regulation of these genes.

### NCL proteins are secreted during *Dictyostelium* development

Many lysosomal enzymes have been detected extracellularly and their roles outside the cell are gaining increased attention with respect to disease development and progression [106]. With that in mind, observations first made in *Dictyostelium* suggest that secretion may play an important role in the pathology underlying the NCLs. Proteomic-based analyses revealed that 5 of the 11 NCL protein homologs are secreted during *Dictyostelium* multicellular development including Ppt1, Tpp1B and Tpp1F, Cln5, CtsD, and proteins that share sequence similarity with human CTSF/CLN13 (CprA, CprB, CprD, CprE, CprF, CprG, uncharacterized protein DDB0252831) [47, 56] (Fig. 2). While CLN5 and CTSD/CLN10 have been detected extracellularly in mammalian systems, the observations in *Dictyostelium* are significant since they provided the first evidence in any system that PPT1/CLN1, TPP1/CLN2, and CTSF/CLN13 may function outside the cell. However, the precise functions of NCL proteins outside the cell are not known. Since they function as enzymes intracellularly, it is possible they may have enzymatic activity outside the cell, but this remains to be determined. In support of extracellular enzyme activity, previous work in *Dictyostelium* detected Tpp1 and CtsD activity in conditioned starvation buffer [94]. However, additional work is required to determine if these activities can be attributed to secreted NCL proteins and if these extracellular activities can be detected in mammalian models of NCL. In support of the molecular networking of NCL proteins, loss of *cln3* alters protein secretion during development, including homologs of NCL proteins (Cln5, CtsD, CprA, CprB, CprD, CprE, CprF, CprG) and lysosomal enzymes such as N-acetylglucosaminidase and cathepsin B [47] (Fig. 2). Secretion of Cln5 and CtsD is also affected by the loss of *mfsd8* [46]. These discoveries indicate that aberrant secretion may play an important role in NCL pathology. In fact, this work has informed research in mammalian systems. For example, aberrant secretion was subsequently reported in glia and neurons isolated from *Cln3*-deficient mice, and brain and cerebrospinal fluid from NCL patients (CLN1, CLN2, and CLN3 disease) [107, 108]. Together, these findings highlight the importance of determining the impact of altered protein secretion in the NCLs.

### Common phenotypes in mammalian and *Dictyostelium* models of NCL

As discussed above, there is evidence at the molecular level linking the NCL proteins to a common biological pathway (e.g., similar localizations, binding and interactome studies, expression analyses). Importantly, these observations are supported by work at the cellular and organismal level, which shows that loss or mutation of individual NCL genes causes similar phenotypes in *Dictyostelium* and mammalian models of NCL. These studies will be discussed below to highlight the possible role of NCL proteins in regulating a common cellular pathway.

#### Mammals

In CLN3 disease patient fibroblasts, the V0a1 subunit of v-ATPase, which regulates lysosomal acidification, is mis-localized to the plasma membrane instead of its normal location on the lysosomal membrane [89]. This defect dysregulates lysosomal acidification, which has also been reported in *Cln1*^*−/−*^ mice. In addition, lysosomal fractions from mouse models of CLN3 and CLN7 disease display altered amounts of lysosomal enzymes [90, 91]. Previous work also showed that the aberrant growth and apoptosis of CLN5 disease patient fibroblasts can be corrected by re-introducing the CLN8 protein [109]. Interestingly, like CLN5 disease patient fibroblasts, ceramide synthase activity is also decreased in *CLN8*^*−/−*^ cells derived from a naturally occurring mouse model for CLN8 disease suggesting that CLN5 and CLN8 may function in the same biological pathway [109]. Additionally, several NCL disease models display aberrant autophagy, which has also been reported in other forms of neurodegeneration [110]. In fact, previous work has linked the functions of 10 of the 13 NCL proteins to autophagic mechanisms (PPT1/CLN1, TPP1/CLN2, CLN3, CLN5, CLN6, MFSD8/CLN7, CTSD/CLN10, PGRN/CLN11, ATP13A2/CLN12, KCTD7/CLN14) [111–120].

Another possibility for the networking of NCL proteins involves the proteins functioning along converging pathways. In mice, loss of both *Cln1* and *Cln5* causes more severe phenotypes and leads to an earlier onset of the disease than in mice where only one of *Cln1* or *Cln5* is knocked out [121]. While fertile, the double knockout mice showed a slight decrease in expected breeding ratios as well as impaired embryoid body formation by induced pluripotent stem cells derived from the mice. These results suggest that the enhanced pathological phenotypes caused by the simultaneous loss of both *Cln1* and *Cln5* may be caused by the disruption or dysregulation of converging pathogenic pathways.

#### Dictyostelium

Several gene-deficiency models have been generated in *Dictyostelium* that have enhanced our knowledge of NCL protein function in this model organism (*tpp1A*^*−*^, *cln3*^*−*^, *cln5*^*−*^, and *mfsd8*^*−*^ cells) (Table 4). Autofluorescent material has been detected in *tpp1A*^*−*^, *cln3*^*−*^, and *cln5*^*−*^ cells and these knockout cells display common phenotypes [44, 48, 94, 122]. For example, *cln3*^*−*^ cells display increased cell proliferation during growth [43]. Loss of *tpp1* also affects cell proliferation, but the effect is reduced growth, not increased, suggesting that while a common growth-regulating pathway may be affected in all three cell lines, the proteins may serve different functions in this pathway [122]. Knockdown of *tpp1* increases phagocytosis and micropinocytosis, the latter of which has also been reported for *cln3*^*−*^ cells [43, 122]. During multicellular development, both *cln3*^*−*^ and *cln5*^*−*^ cells display reduced adhesion [45, 48]. Finally, loss of *tpp1* or *cln3* causes cells to develop precociously and *tpp1* knockdown cells form smaller slugs and fruiting bodies [43, 44, 122]. Together, findings from *Dictyostelium* and mammalian models of NCL suggest that similar downstream secondary events may occur in the cells of NCL patients. However, the specific mechanisms underlying these events in the individual NCL subtypes remain to be determined.

**Table 4:** Gene-deficiency phenotypes in *Dictyostelium* NCL gene knockout models

Phenotype	Cell Line			
	***tpp1A***^***−***^	***cln3***^***−***^	***cln5***^***−***^	***mfsd8***^***−***^
**Storage body accumulation**	Yes	Yes	Yes	Not tested
**Aberrant cell proliferation**	No (Yes, *tpp1* knockdown)	Yes	Not tested	Not tested
**Impaired cytokinesis**	Not tested	Yes	Not tested	Not tested
**Defects in osmoregulation**	Not tested	Yes	Not tested	Not tested
**Impaired autophagy**	Yes	Not tested	Not tested	Not tested
**Reduced chemotaxis**	Not tested	Yes	Yes	Not tested
**Aberrant secretion**	Not tested	Yes	Not tested	Yes
**Reduced adhesion**	Not tested	Yes	Yes	Not tested
**Delayed aggregation**	Not tested	Yes	Not tested	Not tested
**Precocious development**	Yes	Yes	Not tested	Not tested
**Impaired spore formation**	Yes	Not tested	Not tested	Not tested
**Impaired spore integrity**	Not tested	Yes	Not tested	Not tested

### Future avenues for exploring the molecular networking of NCL proteins

Since there is currently only one approved therapy for the NCLs, which is specific for one subtype of the disease, additional work is needed to determine the cellular pathways affected by mutations in NCL genes. As discussed in this review, important information can be obtained by examining the localizations of NCL proteins, assessing their interactions with one another and common binding partners, and examining the effects of individual NCL gene loss or mutation on other NCL genes/proteins. Continued work in these areas is vital for defining the cellular roles of NCL proteins, the pathways they regulate, and the conditions that impair their functions. To supplement this research, future work that systematically examines NCL gene expression in specific models of the disease has the potential to elucidate new interactions between NCL genes/proteins. For example, most of the NCL proteins fall into one of two general classes, enzymes (e.g., TPP1/CLN2, CTSD/CLN10) or putative channels/transporters (e.g., CLN3, MFSD8/CLN7). Therefore, it may be possible to suppress phenotypes in certain subtypes of the disease by modulating the expression of other NCL genes. When considering approaches for treating patients with NCL, we must consider that it may not be possible to restore the normal function of the mutated protein. With that in mind, RNA sequencing can be used to identify cellular pathways affected by NCL gene loss or mutation. A comparison of the datasets from different models can then provide insight into the cellular pathways affected by the NCLs, as well as potentially reveal proteins associated with multiple subtypes of the disease, which may lead to the identification of new molecular targets. Therapies could theoretically be designed against these new targets to potentially suppress clinical symptoms in multiple NCL subtypes.

### Future potential of *Dictyostelium* for NCL research

While mammalian and patient-derived cells have the potential to enhance our understanding of the pathological mechanisms underlying the NCLs, the ability of *Dictyostelium* to inform research in these systems holds much promise, especially since the genome encodes more homologs of NCL proteins than other eukaryotic model organisms (e.g., yeast, worm, and fruit fly) [9]. Future work that studies the localizations of the uncharacterized NCL protein homologs in *Dictyostelium* will broaden our understanding of the pathways regulating NCL protein function and will allow us to identify specific regions of the cell that are affected by NCL. These findings can then be applied to therapy design efforts to determine which parts of the cell need to be targeted in patients. Unlike other model systems, researchers can also exploit the *Dictyostelium* life cycle to assess protein-protein interactions during both growth and multicellular development. This can then lay the foundation for future work that assesses the effects of mutations on the association of NCL proteins with each other, as well as proteins critical to their function.

However, with all that said, no model is perfect, and researchers must consider that *Dictyostelium* does present some limitations as a model organism for NCL research. For one, the limited number of cell types in *Dictyostelium* may limit the translation of findings to specific tissues or organs in mammalian systems. *Dictyostelium* also lacks a central nervous system, so discoveries made in the organism must be evaluated in the relevant mammalian cell type. Nonetheless, *Dictyostelium* presents a useful model system for exploring the functions of NCL protein homologs in a simple eukaryotic organism with measurable phenotypic outcomes.

### Alternate perspectives and considerations

Our current understanding of the mechanisms underlying the NCLs is based on findings from various models ranging from organisms such as *Dictyostelium*, to mice, immortalized mammalian cells, and reprogrammed patient-derived cell lines. However, we must acknowledge that research in these systems may not accurately reflect the situation in patients, especially when one considers the variety of cell types affected by the disease. Multicellular organisms such as *Dictyostelium* may also display cell type-specific effects not observed in mammalian models or patients. With those caveats in mind, conserved findings among multiple systems were highlighted throughout this review to demonstrate their potential significance to NCL pathology. While it is important to acknowledge the caveats of previous work, the findings summarized in this review can be used as a springboard to help guide us into new viable avenues of research.

Finally, the broadly similar clinical and pathological profiles of the different NCL subtypes suggest that common disease mechanisms may be involved, which has been the focus of this review. However, a growing body of evidence also suggests that distinct mechanisms may be implicated in some forms of NCL, which is not surprising given the diversity of processes affected by mutations in NCL genes and the emerging differences in the nature and timing of pathology for the different subtypes [123]. Therefore, future work that explores both these avenues will be necessary to broaden our understanding of the cellular and molecular mechanisms underlying the NCLs.

## Conclusions

The goal of this review was to integrate findings across *Dictyostelium* and mammalian models to shed light on the networking of NCL proteins within the eukaryotic cell. The localizations of NCL proteins suggest they play a concerted role in regulating the trafficking of material within the cell. This is supported by work showing that some NCL proteins bind to each other or have common binding partners. In addition, loss or mutation of individual NCL genes affects the expression and/or activities of other NCL genes/proteins. Finally, genetic models of NCL display similar phenotypes, which provides cellular and organismal evidence potentially linking the NCL proteins to a common cellular pathway. This review also highlighted an important observation; the links between CTSD/CLN10 and half of the NCL genes/proteins. While the precise role of CTSD/CLN10 in the NCLs is not known, that data point toward CTSD/CLN10 possibly being a common pathogenic feature of all NCL subtypes.

The continued use of mammalian and non-mammalian cellular models is vital for providing new insight into the networking of NCL proteins within cells, which in turn will facilitate to the identification of new molecular targets for therapy development. As previously mentioned, recent genetic advances show a strong overlap of some NCL subtypes with later onset forms of neurodegeneration including Alzheimer’s disease, Parkinson’s disease, and frontotemporal dementia. Therefore, the development of new therapies for the NCLs could also be highly beneficial for people affected by other forms of neurodegeneration.

## Data Availability

N/A

## Abbreviations

AprA: Autocrine proliferation repressor A
ATP13A2: ATPase cation-transporting 13A2
Bip2: Luminal-binding protein 2
CadA: Calcium-dependent cell adhesion protein A
CfaD: Counting factor-associated protein D
CLN: Ceroid lipofuscinosis neuronal
Cpr: Cysteine proteinase
CTS: Cathepsin
CV: Contractile vacuole
DBH: Dopamine beta-hydroxylase
DNAJC5: DnaJ homolog subfamily C member 5
ER: Endoplasmic reticulum
FTD: Frontotemporal dementia
GluA: Beta-glucosidase
GPR78: Glucose-regulated protein 78
GPHR: Golgi pH regulator
iPSC: induced pluripotent stem cell
KCTD7: Potassium channel tetramerization domain-containing protein 7
ManA: Alpha-mannosidase
Mcf: Mitochondrial substrate carrier family protein
MEF: Mouse embryonic fibroblast
MFSD8: Major facilitator superfamily domain-containing protein 8
NagA: N-acetylglucosaminidase
NCL: Neuronal ceroid lipofuscinosis
PGRN: Progranulin
PPT1: Palmitoyl protein thioesterase 1
TPP1: Tripeptidyl peptidase 1
