# Correction to: Molecular networking in the neuronal ceroid lipofuscinoses: insights from mammalian models and the social amoeba *Dictyostelium discoideum*

**DOI:** 10.1186/s12929-020-00686-3

**Published:** 2020-09-21

**Authors:** Robert J. Huber

**Affiliations:** Department of Biology, Trent University, 1600 West Bank Drive, Peterborough, Ontario K9L 0G2 Canada

**Correction to: J Biomed Sci 27, 64 (2020)**

**https://doi.org/10.1186/s12929-020-00653-y**

In the original publication of this article [1], the word ‘biologys’ in the sentence ‘Therefore, *Dictyostelium* presents an excellent system to dissect the roles of SLC25A1 and SLC25A13 in NCL biologys.’ on Page 8 is incorrect. The correct sentence should be ‘Therefore, *Dictyostelium* presents an excellent system to dissect the roles of SLC25A1 and SLC25A13 in NCL pathology.’
